# Mitochondrial DNA and the largest nuclear-mitochondrial DNA in Arabidopsis can be separated by their methylation levels

**DOI:** 10.1093/plphys/kiaf069

**Published:** 2025-02-20

**Authors:** Yuyang Zhong, Miki Okuno, Nobuhiro Tsutsumi, Shin-ichi Arimura

**Affiliations:** Laboratory of Plant Molecular Genetics, Graduate School of Agricultural and Life Sciences, The University of Tokyo, Tokyo 113-8657, Japan; Division of Microbiology, Department of Infectious Medicine, Kurume University School of Medicine, Fukuoka 830-0011, Japan; Laboratory of Plant Molecular Genetics, Graduate School of Agricultural and Life Sciences, The University of Tokyo, Tokyo 113-8657, Japan; Laboratory of Plant Molecular Genetics, Graduate School of Agricultural and Life Sciences, The University of Tokyo, Tokyo 113-8657, Japan

## Abstract

Methylation of cytosines in plant mitochondrial DNA (mtDNA) has been a controversial issue. Results supporting mtDNA methylation may have been subject to contamination due to the presence of nuclear sequences originating from the mitochondrial genome called nuclear mitochondrial insertions (NUMT). In Arabidopsis (*Arabidopsis thaliana*) Columbia 0 (Col-0), the largest NUMT, located on Chromosome 2, is nearly twice the size of the entire mitochondrial genome and exhibits a sequence almost identical to the mitochondrial genome, albeit with shuffling and repeats. In the presence of such high similarity, it is challenging to eliminate interference when determining mtDNA methylation levels. Here, we applied a methyl-CpG-binding domain (MBD) protein-based affinity assay to separate total DNA, applied next-generation sequencing to the pre- and postseparation DNA samples, and examined the single nucleotide polymorphism (SNP) sites between NUMT and mtDNA. The results revealed successful separation of methylated and non-methylated DNA within the total DNA, with simultaneous separation achieved between NUMT DNA and mtDNA. These results suggest that our method can achieve separation based on the differential methylation levels of the whole lengths of NUMT and mtDNAs. The bisulfite sequencing results for the postseparation DNA samples suggest that mtDNA exhibits not only a lack of CpG methylation but also an absence of CHH and CHG methylation. In contrast, the NUMT shows high levels of methylation across all 3 contexts, at least in the Col-0 accession.

## Teaser

Mitochondrial DNA (mtDNA) and the 641 kb nuclear mtDNA in Arabidopsis Columbia 0 (Col-0) can be clearly separated during affinity precipitation by the absence and presence of cytosine methylation levels.

## Introduction

As one of the epigenetic modifications, DNA methylation plays a crucial role in the physiological processes of organisms (Moore et al. 2013; Zhang et al. 2018). Mitochondrial DNA (mtDNA) methylation in mammals, fungi, and plants has been explored so far (Shock et al. 2011; Kang et al. 2017; Muniandy et al. 2020), but it is still controversial. Some studies challenge the perspective on human and mouse mtDNA methylation (Mechta et al. 2017; Bicci et al. 2021). In Arabidopsis (*Arabidopsis thaliana*), ecotype Columbia 0 (Col-0), the mtDNA methylation and its level change are reported (Wang et al. 2022). It is worth noting that the presence of probable DNA-methylated nuclear mitochondrial insertions (NUMTs) (Noutsos et al. 2005; Wei et al. 2022) was not taken into account in this study. In Col-0, nearly the entire mitochondrial genome has been inserted into the pericentromeric region of Chromosome 2, forming the largest NUMT in the nuclear genome (Fields et al. 2022). Not only is this NUMT larger than the entire Col-0 mitochondrial genome (641 kb vs 367 kb), but it also shares 99.933% sequence identity (Supplementary Fig. S1) with the mitochondrial genome and would be highly methylated. Therefore, to see the fine DNA methylation levels of Col-0 mtDNA, contamination from NUMT DNA sequences should be carefully considered.

Another problem in detecting DNA methylation is the commonly used methods, including bisulfite conversion-based methods such as whole genome bisulfite sequencing (Gu et al. 2011), affinity enrichment-based methods such as methylated DNA immunoprecipitation (MeDIP or mDIP) (Zhao et al. 2014), and restriction enzyme (RE)-based methods such as comprehensive high-throughput arrays for relative methylation (CHARM) (Irizarry et al. 2008). These assays often require the digestion of long sequences into shorter sequences before they can be read and detected. When using any of these methods, it is essential to take careful precautions to avoid artifacts where methylation of DNA from NUMTs is mistakenly counted as methylation of mtDNA. Even though bisulfite sequencing serves as the gold standard for cytosine methylation measurement, there is still the issue of incomplete conversion of unmethylated cytosine to uracil (Matsuda et al. 2018).

Although NUMT also poses a substantial interference to the determination of mtDNA methylation, it is fortunate that high-quality NUMT sequence information has already been published, indicating the biggest NUMT on Chromosome 2 in Col-0 is highly methylated at 5-methylcytosine (5mC) (Fields et al. 2022). Given that hypomethylation/partial methylation of mtDNA may affect gene expression, genomic stability, and metabolic responses, it is crucial to first ascertain the overall methylation status of mtDNA to thoroughly evaluate the biological significance of mtDNA methylation. In this research, we aim to investigate (i) whether mtDNA in Col-0 is methylated at 5mC, (ii) which parts (and at what developmental stages) of the mtDNA in Arabidopsis Col-0 are 5mC methylated by circumventing NUMT-induced interference. During the course, we found that (iii) the substantial difference in the methylation levels between mtDNA and the NUMT makes it feasible to clearly and easily separate these 2 DNAs from the total DNA with an MBD (methyl-CpG binding domain) protein-involved separation method.

## Results

### The CpG methylation status of NUMT and mitochondrial sequences by using SNPs between them

**Figure 1.**
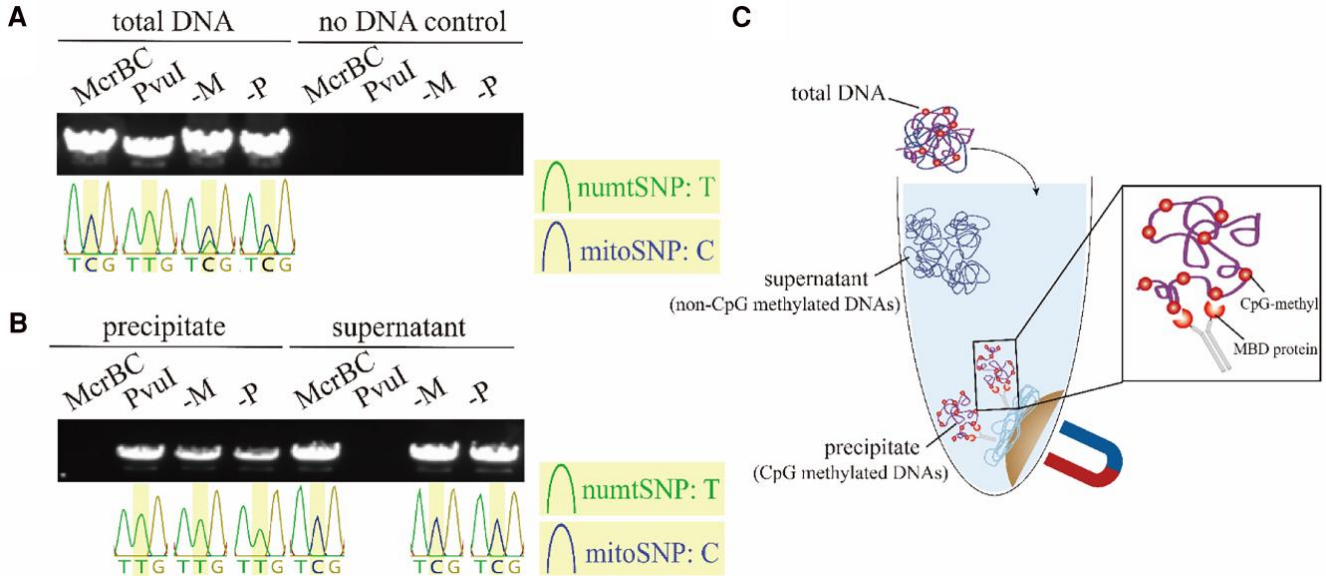
The separation and amplification result on the *rps3* gene region. **A)** and **B)** The amplification of total DNA, the supernatant, and the precipitate portion on the *rps3* gene region. The base in the middle (yellow shade) of the PCR-direct Sanger sequencing chromatogram peak result represents the SNP sites. McrBC, CpG-methylation-dependent RE. *Pvu*Ⅰ, CpG-methylation-sensitive RE. numtSNP: the type of SNP on NUMT; mitoSNP: the type of SNP on mitochondria. -M: McrBC control, no enzyme, only the buffer of McrBC was used; -P: *Pvu*Ⅰ control, no enzyme, only *Pvu*Ⅰ buffer was used. **C)** The rationale of MBD protein-binding precipitation.

Plant DNA cytosine methylation occurs in the CG (55%), CHG (23%), and CHH (22%) contexts in the nucleus (Lister et al. 2008). In this study, we primarily focus on the CG context in mtDNA. The CpG-methylation-sensitive RE *Pvu*Ⅰ and the CpG-methylation-dependent RE McrBC (Sutherland et al. 1992) have been used in this case. Total DNA from leaves of Col-0 (1-mo-old) was first extracted and digested by these 2 REs, and then amplification was applied to the ribosomal protein S3 (*rps3*) gene area shared by both mitochondrial and NUMT sequences. Sanger sequencing to identify the single nucleotide polymorphism (SNP) at +1904C/T in the *rps3* region between mitochondrial and NUMT sequences was applied to the amplification products (Fig. 1A). In this RE and amplification assay, in both non-RE treatment controls (-M, -P), the overlap of NUMT-type SNPs (numtSNPs) and mitochondrial-type SNPs (mitoSNPs) in the Sanger sequencing chromatogram peaks was observed. After the McrBC treatment, only mitoSNP remained, suggesting that all the NUMT *rps3* sequences seemed to be digested, and mtDNA remained to be amplified. As for *Pvu*Ⅰ treatment, only the numtSNP remains, suggesting that mtDNA in the targeted *rps3* area was digested by *Pvu*Ⅰ so that it cannot be amplified. This result indicates a lack of CpG-methylation, at least in the *rps3* area of the mitochondrial genome, but the complete CpG methylation of the corresponding NUMT *rps3* area.

Next, we used an MBD protein-based affinity precipitation assay to separate the CpG-contained DNA and others from the total DNA. The MBD protein can only bind to the CpG-methylated DNA sequences (Nan et al. 1993); therefore, when total DNA was put into the MBD protein-involved system, the CpG-methylated DNA was captured and separated (Fig. 1C). Similar RE and amplification assay was applied to the precipitate and the supernatant samples (Fig. 1B). In the precipitated sample, all sequences were digested by McrBC, suggesting the high CpG methylation in this portion; whereas in the supernatant sample, all the sequences were digested by *Pvu*Ⅰ, suggesting an absence of CpG methylation in this portion. Taken together, the separation of CpG-methylated DNA containing and non-CpG-methylated DNA was achieved by this MBD protein-based assay. Furthermore, in the precipitated samples, the SNPs of RE and no-RE treatment were both numtSNP, suggesting that the precipitate part contains exclusively NUMT. For the supernatant sample, the SNPs of RE and no-RE treatment were mitoSNPs, suggesting that the supernatant part contains exclusively DNAs from mitochondria. Collectively, the precipitated sample is highly CpG-methylated and contains DNA from NUMT, at least in the *rps3* region (the methylation status of other areas can be drawn from next-generation sequencing [NGS] data in the later part), while the supernatant sample is absent from CpG methylation and contains DNA of the corresponding area from mitochondria. This result hints that the MBD protein-based affinity precipitation assay allows for the separation of mtDNA and NUMT.

**Figure 2.**
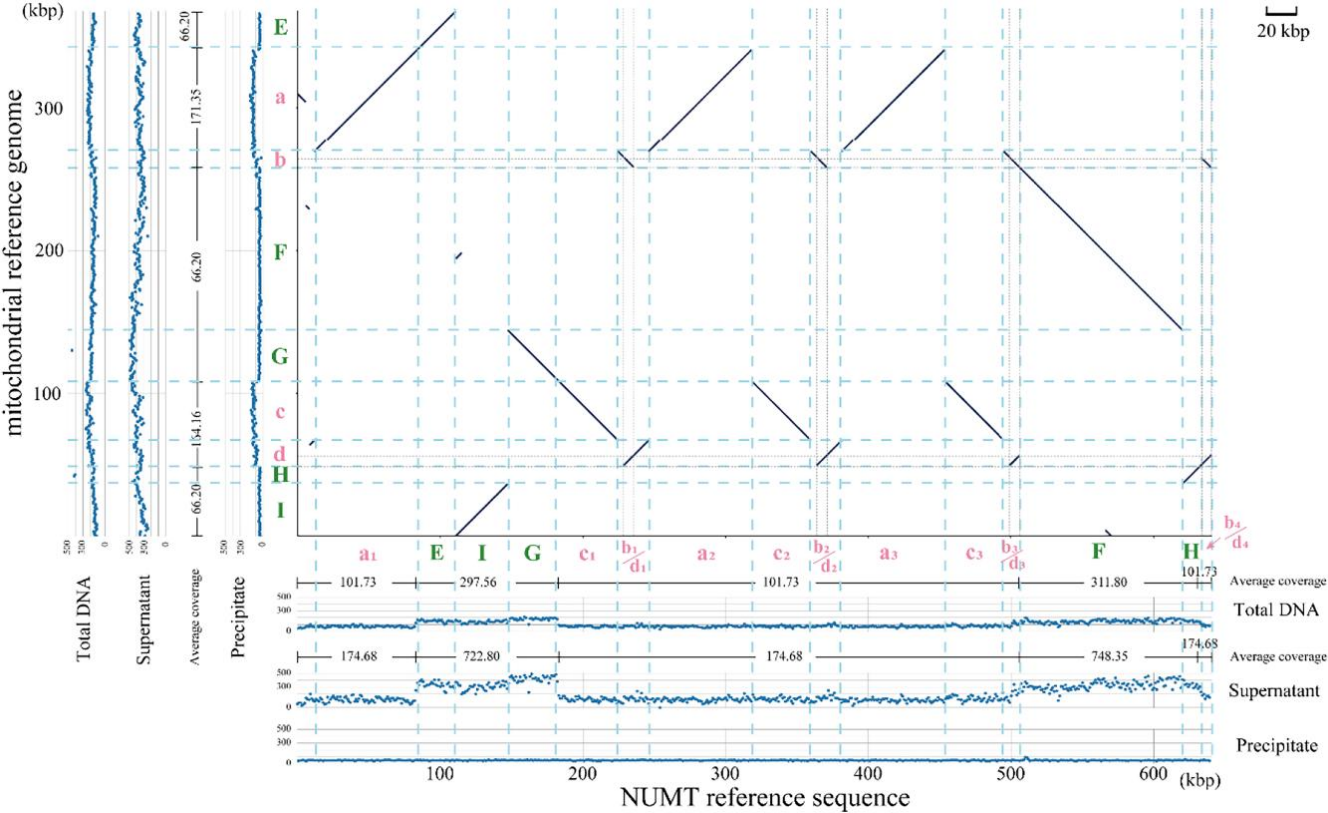
Dot-plot alignment between the mitochondrial genome and the NUMT sequence, and each of the read depth distributions of the WGS data. The horizontal axis shows the NUMT region in Chromosome 2 (Col-CEN) and the vertical axis shows the mitochondrial reference genome (BK010421). The lines in the dot-plot represent the aligned regions between the 2 sequences with an identity exceeding 98%. The scatter diagrams to the left and bottom of the dot-plot show the read depth distributions of 3 samples mapped separately for each sequence. Multicopy loci are indicated by pink lowercase letters (a to d in the mitochondrial region, and a_1_ to a_3_, c_1_ to c_3_, b_1_/d_1_ to b_4_/d_4_ in the NUMT region, while single-copy loci are marked with green uppercase letters (E to I in both regions). Supernatant: distribution of read depth of supernatant portion mapping to the corresponding reference sequence; Precipitate: distribution of read depth of precipitate portion mapping to the corresponding reference sequence. The average coverage values for the regions enclosed by the horizontal and vertical lines have been calculated and are marked in the center of each region.

To see the rough CpG-methylation status of the over the whole length of the mtDNA and NUMT, Illumina-based NGS was applied to the total DNA, precipitated DNAs, and supernatant DNAs. The short reads from these 3 DNA samples were then mapped to the NUMT reference sequence or the mitochondrial reference genome. Their read coverages and a dot-plot analysis, which showed the sequence similarity between them, are combinedly shown in Fig. 2. Additionally, a synteny plot providing a more intuitive representation of the similar regions between the NUMT and mtDNA sequences has been added to Supplementary Fig. S1. Because of the high similarity between the NUMT and mitochondrial genome sequences (99.933%), reads from the NUMT can be mapped to the mitochondrial sequence and vice versa. Here, we expected to determine the origin of the reads, whether from NUMT or from mtDNA, through coverage patterns. The coverage pattern can be either flat, indicating that the reads are mapped to the original sequence, or shown “steps”, indicating that the reads are mapped to a similar but different-derived sequence with differences in copy number (repeats) across certain regions. The dot-plot shows the regions with different copy numbers or repeats, between the mitochondrial genome and the NUMT sequence. The coverage pattern of short reads from the precipitated DNA mapped to the NUMT reference sequence looked overall flat over the whole length. On the contrary, the precipitated DNA sample aligned to the mitochondrial reference genome, which contains mainly 4 regions (horizontal axis, a to d, in pink lower case, “steps”) of the 3 to 4 times the increased number of reads mapped were observed. The dot-plot shows that these 2 regions correspond to the 3 (or 4) times repeated sequences in the NUMT (vertical axis, a_1_ to a_3_, c_1_ to c_3_, b_1_/d_1_ to b_4_/d_4_). This implies that almost all the reads from the precipitate portion are derived from the NUMT. Similarly, the supernatant reads were mapped roughly flatly to the mitochondrial genome, and the reads from supernatant portions mapped to the NUMT reference genome contained 5 special areas (vertical axis, E to I, in green upper case, “steps”) of the 3 to 4-times increased number of reads were also observed. This suggests that the supernatant reads are almost all from the mtDNA genome. The coverage patterns of total DNA showed an intermediate but slightly more similar to the pattern of the supernatant, which seems reasonable because the copy number of the mtDNA is much higher than the nuclear DNA in the cells (Preuten et al. 2010; Wang et al. 2010). Collectively, the whole lengths of NUMT and mtDNA seem separated clearly by the MBD protein-based affinity precipitation assay.

**Figure 3.**
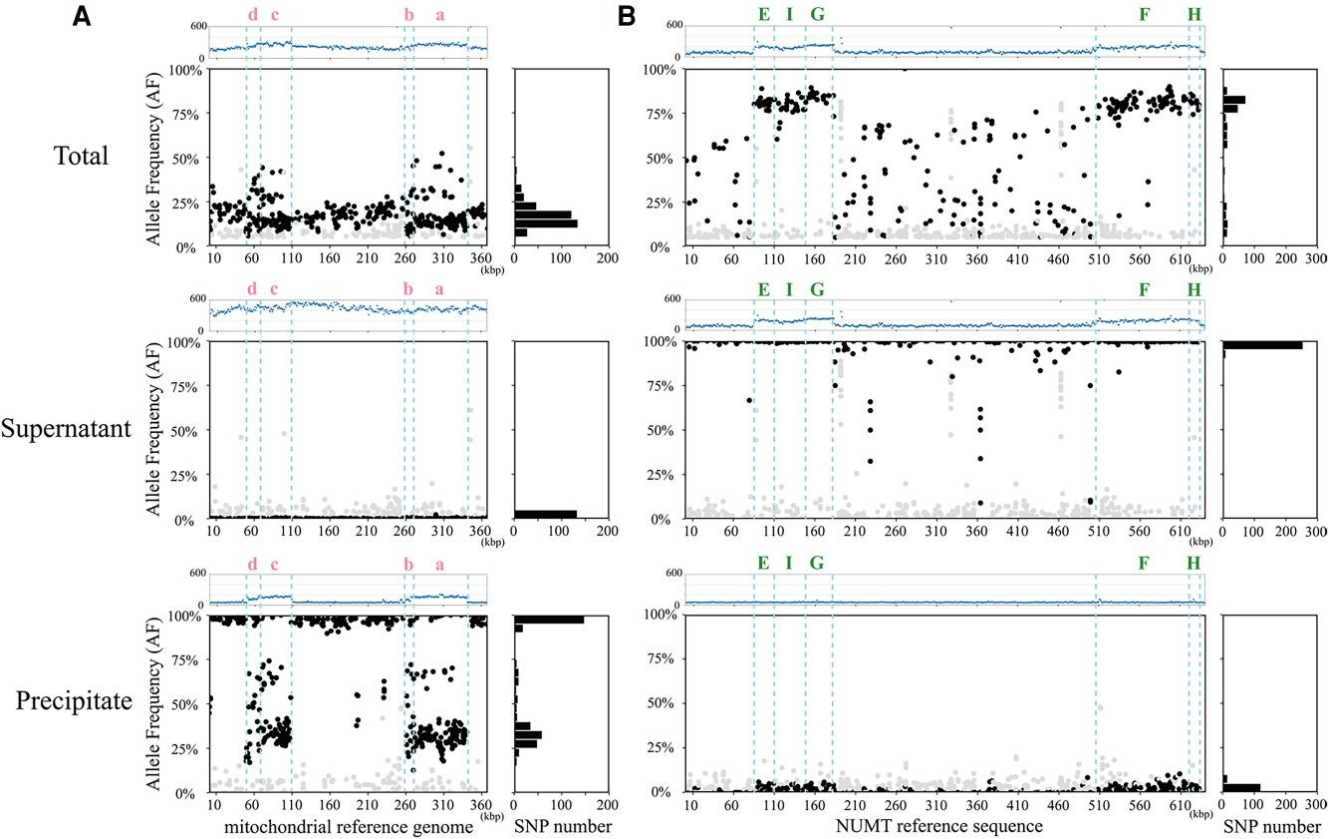
SNP calling and AF calculation of NGS reads from total, supernatant, and precipitate DNA samples. Reads of total DNA, supernatant, and precipitate DNA samples were mapped to **A)** mitochondrial reference genome and **B)** NUMT reference sequence, respectively. The black scatter diagrams show the allele positions and frequencies of the SNPs between mitochondrial and NUMT reference sequences, the gray scatters represent sequencing errors and interference reads from the chloroplast and NUMT outside Chromosome 2. The blue scatter diagrams at the top of each figure represent the read depth. The total number of SNPs between mitochondrial and NUMT reference sequences for every 5% of allele frequency interval is shown by the bar plots on the right side of each figure. The regions marked by pink lowercase letters correspond to the multicopy loci in Fig. 2, while the regions marked by green uppercase letters correspond to the single-copy loci in Fig. 2.

To see the origin of the reads mapped to each of the reference sequences in a more detailed and comprehensive view, after mapping NGS reads from the total DNA, supernatant DNAs, and precipitate samples to the mitochondrial reference genome or to the NUMT reference sequence, the allele frequency (AF) at each SNP position was calculated by dividing allelic depth (AD) by the total depth of coverage (DC), representing the ratio of reads originating from the precipitate and supernatant samples (Fig. 3, Supplementary Fig. S2). AD here refers to the number of reads with SNPs at a given locus that differs from the reference genome, while DC represents the total number of reads mapped at that locus. When AF equals 100%, it means that all the reads differ from the reference sequence, whereas when AF equals 0%, it means that all the reads are identical to the reference sequence. The SNP sites between mitochondrial and NUMT reference sequences are highlighted in the black dots in Fig. 3, and the gray scatters represent sequencing errors and interference reads from chloroplast or NUMT sequences outside Chromosome 2. Therefore, the following discussion focuses on only the black dots.

A total of 381 SNP sites were found when mapping the reads from the total DNA portion to the mitochondrial reference genome (Fig. 3A, top), with the majority concentrated within the 10% to 20% AF range. This means that (i) the mixture of reads from both mtDNA and NUMT DNA was mapped to the mitochondrial reference genome sequence, and (ii) the reads of mtDNA are more than that of the nuclear genome, which is due to the difference of copy numbers in each cell (Wang et al. 2010). When mapping reads from the supernatant portion to the mitochondrial reference genome (Fig. 3A, middle), the number of SNP sites decreased, with only 133 identified, and the AF values of most SNPs decreased to within 5%. This means the non-CG-methylated DNA portion of total DNA includes mainly (almost all) mtDNA but contains minimal NUMT DNA (numtDNA). When mapping reads from the precipitate portion to the mitochondrial reference genome, 381 SNP sites were found and the values of AF are primarily focused within the range of 95% to 100% (Fig. 3A, bottom). In the multicopy loci regions (indicated by pink lowercase letters, a to d), clusters of black scatter points with AF values ranging between 25% to 40% and 60% to 70% were also observed. These intermediate AF values were largely due to SNPs between the copied (repeat) regions in the NUMT. For example, the open reading frame 262 (*orf262*) gene region in mitochondria has been inserted into 3 areas in NUMT (Supplementary Fig. S3). In most cases, SNPs are located on only 1 of the 3 copies, but sometimes SNPs are located on more than 1 copy. Based on the high AF values for the reads of the precipitate portion mapped to the mitochondrial reference genome, and the alignment of the black scatter point clusters with the repeated insertion regions, this indicates that the precipitate part contains almost no reads from mtDNA, but those from the NUMT.

A total of 287 SNP sites were found, and most of them were concentrated within 75% to 85% AF when mapping reads from the total DNA portion to the NUMT reference sequence (Fig. 3B, top). Meaning that (i) reads from both mtDNA and NUMT DNA were mapped to the NUMT reference sequence, and (ii) most of the SNP sites concentrated within 75% to 85% AF reflect that more reads from mtDNA than NUMT were mapped to the NUMT reference sequence. A total of 286 SNP sites were identified when mapping the supernatant portion to the NUMT reference sequence (Fig. 3B, middle). The majority of these SNPs were concentrated in the 95% to 100% range, indicating that most reads from the supernatant portion are derived from mtDNA, with almost no NUMT DNA present in this portion. For the precipitate portion mapped to the NUMT reference sequence, 138 SNP sites were identified, with most of the SNPs having AF values in the range of 0% to 5%. This means that the CpG-methylated portion includes mainly NUMT DNA. Compared to the reads from total DNA mapped to the precipitate portions, nearly half of the SNP sites disappeared, suggesting the precipitate portion exhibits high similarity with the NUMT reference sequence. The multicopy loci, indicated by lowercase letters in Fig. 2, and the single-copy loci, indicated by uppercase letters, are similarly marked in Fig. 3 and Supplementary Figs. S1 and S2. The multicopy loci regions exhibit a greater diversity of AF values. Due to the similarity between the reads from the supernatant portion and the mitochondrial reference genome sequence, the supernatant portion contains almost exclusively mtDNA. As those from the precipitated portion resemble the NUMT reference sequence, the precipitated portion contains almost exclusively numtDNA. Taken together, mtDNAs and numtDNAs were almost non-CpG-methylated and CpG-methylated over the whole lengths, respectively, and they could be separated through the MBD protein-based affinity precipitation assay.

### The cytosine methylation percentage in precipitate and supernatant portion at CpG, CHH, and CHG context

**Figure 4.**
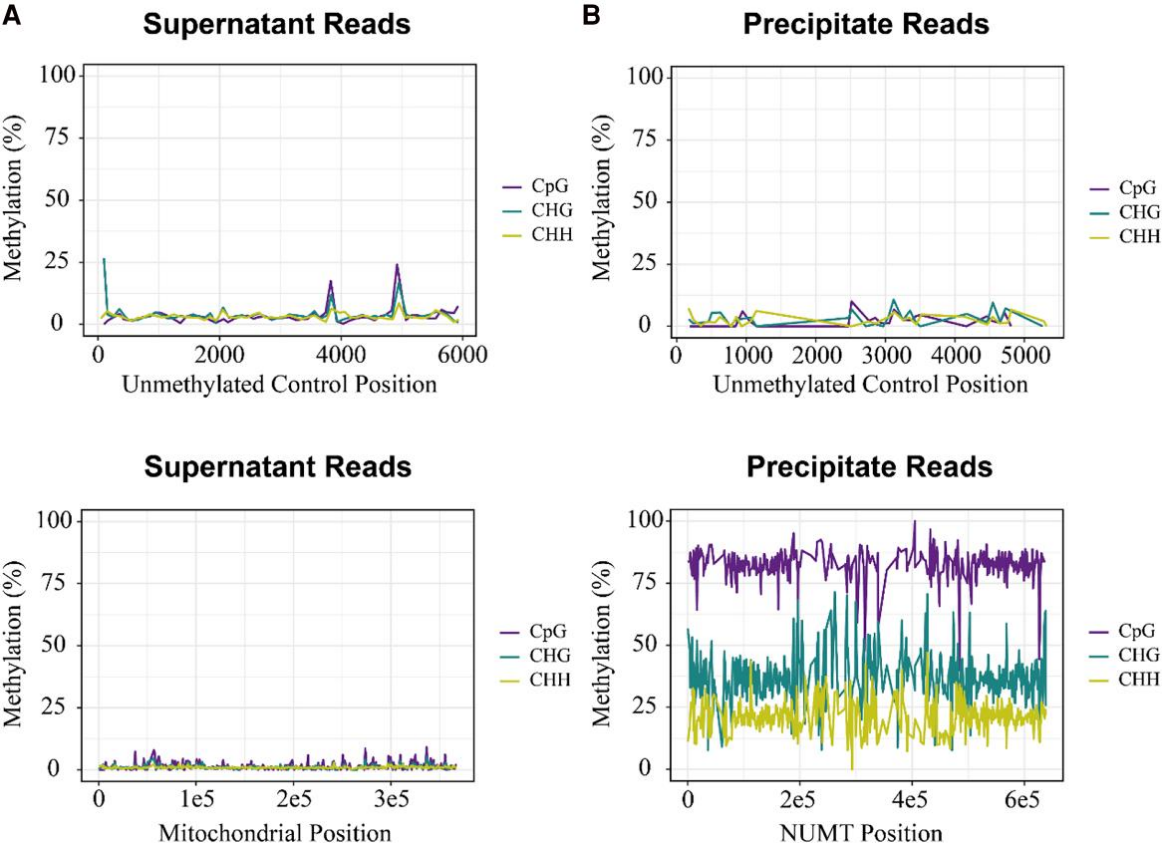
The estimated methylation percentage of unmethylated control, supernatant, and precipitate samples in CpG (darkorchid), CHG (cyan), and CHH (yellow) contexts. **A)** Reads from the supernatant samples were mapped to an unmethylated control reference sequence. The methylation profile was averaged over 100 bp windows. Reads from the supernatant portion were also mapped to the mitochondrial reference genome, and the methylation profile was averaged over 1 kb windows. **B)** Reads from the precipitate portion were mapped to unmethylated control reference sequence. The methylation profile was averaged over 100 bp windows. Reads from the precipitate portion were also mapped to the NUMT reference sequence. The methylation profile was averaged over 1 kb windows.

To confirm the CpG, CHH, and CHG methylation levels in the NUMT and mitochondrial genome, the precipitated and supernatant samples were subjected to bisulfite sequencing. To avoid bisulfite sequencing errors, we amplified a 5,976 bp PCR-generated unmethylated sequence as a control, which was then mixed with the supernatant and precipitate samples separately before bisulfite conversion and sequencing. The bisulfite sequencing reads of the supernatant portion were mapped to the unmethylated control and mitochondrial reference sequence, respectively (Fig. 4A). The methylation level is calculated by dividing the number of reads with C bases (unconverted to T) at a specific cytosine site by the total number of reads aligned to that site. For the unmethylated control in the supernatant portion, an average of 2.93% CpG methylation, 3.33% CHG methylation, and 3.08% CHH methylation were detected. As for supernatant portion mapping to the mitochondrial reference genome, an average of 1.19% CpG methylation, 1.01% CHG methylation, and 0.87% CHH methylation were detected. This indicates that mtDNA is not only devoid of CpG methylation but also lacks CHG and CHH methylation.

Reads from the precipitate portion were mapped to the unmethylated control, NUMT reference sequence, respectively (Fig. 4B). For reads mapped to the unmethylated control reference sequence, an average of 3.71% CpG methylation, 3.45% CHG methylation, and 2.90% CHH methylation was detected. For precipitate reads mapped to the NUMT reference sequence, an average of 82.02% CG methylation, 35.89% CHG methylation, and 21.28% CHH methylation was detected. This echoes the previous nanopore sequencing methylation result (Fields et al. 2022). Taken together, mtDNA is barely methylated in either of 3 methylation contexts, whereas NUMT is highly methylated in all 3 cytosine methylation contexts.

### The CpG methylation status of different tissues and through the time course of growth in Col-0 mtDNA

**Figure 5.**
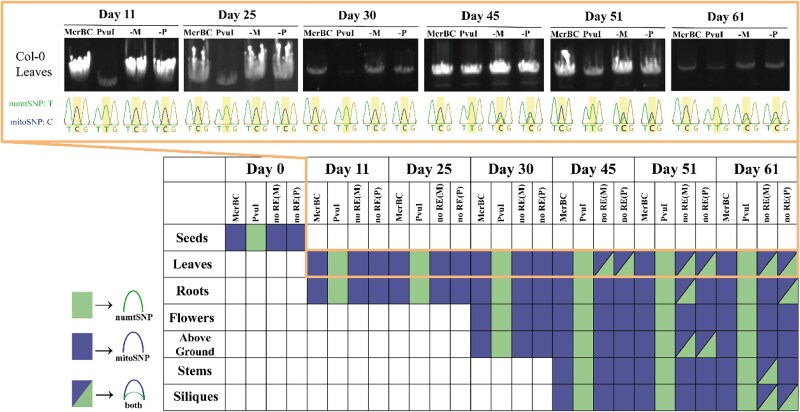
Sanger sequencing results of the *rps3* area amplification with DNA extracted from different tissues and through the time course of growth. For the picture in the orange frame on the top, the base in the middle (yellow shade) represents the SNP sites. McrBC, CpG-methylation-dependent RE. *Pvu*Ⅰ, CpG-methylation-sensitive RE. numtSNP: the type of SNP on NUMT; mitoSNP: the type of SNP on mitochondria. -M: McrBC control, no enzyme, only the buffer of McrBC was used; -P: *Pvu*Ⅰ control, no enzyme, only the buffer of *Pvu*Ⅰ was used. The green rectangle represents the numtSNP and the blue rectangle represents the mitoSNP. The white rectangles mean no data/not examined. The half-blue and half-green rectangles convey the existence of both SNPs.

A similar RE and amplification assay was carried out on DNA from different tissues at different developmental stages of growth in Col-0. No mitoSNP showed through the time course under *Pvu*Ⅰ treatment and no numtSNP was observed throughout the time course under McrBC digestion, indicating the absence of mtDNA methylation at least on the *rps3* gene area across various Arabidopsis Col-0 tissues and growth stages tested here (Fig. 5). In terms of Col-0 leaf mtDNA in the orange frame of Fig. 5, the results of Sanger sequencing indicate that within the group without REs, mitoSNPs were predominantly detected in the first 30 d. As the leaves age, both types of SNPs emerged. This implies that the mitochondrial copy number ratio in young leaves may exceed that in older leaves, resulting in a very low proportion of numtSNPs that cannot be detected in young leaves (Preuten et al. 2010; Zhang et al. 2023). Taken together, no CpG methylation change was detected throughout the time course of DNA extracted from different tissues in Col-0 under our conditions.

## Discussion

Plant mtDNA methylation has been assessed before, relying mainly on methylation-sensitive restriction endonucleases to detect the methylation of mtDNA of wheat embryos and carrots (Bonen et al. 1980; Simkova 1998). Bisulfite sequencing has been considered the gold standard for detecting DNA methylation (Frommer et al. 1992; Gu et al. 2010; Muniandy et al. 2020), which involves the fragmentation of long DNA before sequencing. Additionally, the linearization and ultrasonic fragmentation prior to bisulfite conversion were shown to influence the final assessment of human mtDNA methylation levels (Guitton et al. 2022). Meanwhile, a study found that even in artificially synthesized unmethylated DNA, the conversion rate from cytosine to uracil after bisulfite treatment still cannot reach 100% (Matsuda et al. 2018).

Col-0 is still the golden standard ecotype of the model plant Arabidopsis, also widely used in organelle studies. In Col-0, the presence of a NUMT that is larger than the entire mitochondrial genome and has high sequence identity introduces significant challenges in accurately determining DNA methylation within the mitochondrial genome. Fortunately, the high-quality nucleotide sequence of the NUMT on Chromosome 2 serves as a significant rescue to carry out the methylation study because we could get the information of SNPs between the mitochondrial and the almost identical NUMT sequences (Fields et al. 2022). In this study, the RE check suggested a lack of CpG methylation in Col-0 mtDNAs, and the high CpG methylation of corresponding nuclear genome NUMT of *rps3* area. The rudimentary appraisal through 2 types of REs on DNA from different tissues and different developmental stages of Col-0 also failed to detect CpG methylation on the *rps3* gene region in the mitochondrial genome. In plants, cytosine is usually methylated in CG, CHG, and CHH contexts (Lister et al. 2008), yet the utilization of MBD protein in this study limited the examination of methylation within the CG context. NGS sequencing further validated that this affinity precipitation method can effectively separate mtDNA from NUMTs to a significant extent via the difference in cytosine methylation level over the whole length. It was observed that MBD protein preferentially binds to CpG-methylated areas with high density (Zhang et al. 2006). The proportion of reads originating from different portions allows for an inference of the separation efficiency of the MBD protein-involved assay between mtDNA and NUMT. Previous studies (Preuten et al. 2010; Barow and Jovtchev 2007) have indicated that in rosette leaves, due to extensive endoreduplication, nuclear DNA content can reach ∼13C. It is important to note that the extent of endo-reduplication and resulting nuclear DNA content can vary depending on the leaf developmental stage, environmental conditions, and growth factors. The copy number of mitochondria also varies across different developmental stages (Wang et al. 2010). These variabilities could influence the relative copy numbers of organellar DNA and nuclear DNA in different contexts. The result of this study indicated that the MBD protein does not bind to DNA from mitochondria. Therefore, it is suggested that there is no high-density CpG methylation in the mtDNA of Col-0. Moreover, this result also conveys the high CpG methylation in NUMTs. Bisulfite sequencing further confirmed the methylation levels of mtDNA and numtDNA across different methylation contexts. The estimated methylation percentage of mtDNA was notably low in all 3 cytosine methylation contexts, similar to that of the unmethylated control sequences generated by PCR and subjected to the same bisulfite sequencing procedure. This result suggests that mtDNA is not only deficient in CpG methylation but also lacks CHG and CHH methylation. In contrast, bisulfite sequencing of the precipitate portion revealed that NUMT is highly methylated across all 3 methylation contexts.

The potential hypomethylation and portion-specific methylation of mtDNA may influence transcription and respiration-related biological processes. There have been limited reports that clearly reveal whether plant mtDNA is methylated, particularly when considering the interference from the NUMT sequences. In some studies (Simkova 1998; Muniandy et al. 2020; Wang et al. 2022), mtDNA has been detected as partially methylated; however, these studies have not ruled out the possibility that this methylated DNA originated from NUMT. Therefore, to elucidate the functional role of mtDNA methylation, we must first address the question of whether global or partial mtDNA methylation exists by isolating mtDNA from NUMT. Our MBD protein-involved separation assay provides a promising method for distinguishing mtDNA from NUMT contamination. The experimental results clearly demonstrate that, at least under the conditions of this study, the mtDNA of Col-0 is almost not methylated. In future analyses of mtDNA methylation in Col-0, NUMT contamination poses the greatest risk and should be avoided.

## Materials and methods

### Plant growth conditions and DNA extractions

The Arabidopsis (*A. thaliana*) Col-0 was disinfected for 20 min with a solution (9% sodium hypochlorite, 2% Triton) and washed with sterile water. Then, evenly dispense onto 1/2 mS medium with 1% agarose and vernalize at 4 °C in the dark for 7 d. After vernalization, the plants were transferred to the incubator for 14 d, then transplanted in Jiffy pots (Jiffy-7 Peat Pellets, http://www.jiffypot.com/) and cultivated under 22°C, 50 to 150 *μ*mol m^−2^ sec^−1^ light and 16 h light/8 h dark conditions until a size suitable for DNA extraction on 30 d after vernalization. DNA was extracted through the DNeasy Plant Pro kit and Maxwell RSC Plant DNA kit following the manufacturer's instructions.

### Restriction enzyme treatment

Methylation-dependent RE McrBC from TaKaRa (Code:1234A) and methylation-sensitive RE *Pvu*Ⅰ from NEW ENGLAND BioLabs (R3150S) were used, with a reaction system following the manufacturer's instructions. DNA was treated under 37℃ for 90 min. After that, the temperature was raised to 80℃ for 20 min to inactivate enzymes.

### PCR analyses

PCR was carried out with KOD One PCR Master Mix -Blue- (TOYOBO), amplified on ribosomal protein S3 (*rps3*) gene area, using the primers: Fw 5′- CACTGAGGGGAAGGTTGGTC -3′ and Rv 5′- GCTTTGCTACCGGGCTTCTA -3′. DNA after RE treatment was used as PCR templates. PCR program was set according to the primer Tm and product size and run for 25 to 26 cycles.

### MBD protein-based affinity precipitation

The MBD2 (methyl-CpG-binding domain protein 2) protein-involved immunoprecipitation was applied through the NEBNext Microbiome DNA Enrichment Kit (New England Biolabs, USA) by following the manufacturer's instructions. Total DNA was separated into precipitate and supernatant portions. AMPure XP (Beckman Coulter, Product No: A63882) was applied to these 2 portions for purification.

### Next-generation sequencing and data analysis

The purified precipitate and supernatant portion and total DNA were applied to NGS sequencing. Macrogene (Japan) prepared libraries with dual indexing strategy and performed sequencing on an Illumina NovaSeq6000 platform (150 bp paired-end, 4 Gb data). Low-quality and adapter sequences were trimmed from the raw reads using the fastp (Chen et al. 2018). We mapped the trimmed reads to the mitochondrial genome and the NUMT region, respectively, using the bwa-mem2 (https://github.com/bwa-mem2/bwa-mem2), and filtered out inadequately mapped reads with mapping identities ≤97% or alignment coverage rates ≤80% using samtools (https://github.com/samtools/samtools). SNP calling was completed by vcftools (https://github.com/vcftools/vcftools). Data visualization was performed using the “ggplot2” package from R. Figure layout was conducted using Adobe Illustrator. Sequence alignments between the mitochondrial genome and the NUMT region were obtained and visualized using mummer4. And we obtained SNPs between the 2 reference sequences using show-snps after filtering low-quality alignments using the delta-filter (-l 3000, -i 90).

### Bisulfite sequencing and data analysis

The purified precipitate and supernatant portion were applied to bisulfite sequencing. Macrogene (Japan) prepared libraries with dual indexing strategy and performed sequencing on an Illumina NovaSeq_X_Plus platform (151 bp paired-end, 1 GB data for the supernatant portion, and 4 GB data for the precipitate portion). Accel-NGS Methyl-Seq DNA Library EZ DNA Methylation-Gold (Zymo Research) Kit was used as library kit. Quality assessment of the raw pair-end bisulfite sequencing data was performed using FastQC (http://www.bioinformatics.babraham.ac.uk/projects/fastqc/). Low-quality and adapter sequences were trimmed from the raw sequencing data using Trim Galore (https://www.bioinformatics.babraham.ac.uk/projects/trim_galore/). Trimmed paired-end reads were aligned to the prepared reference genome using Bismark (Krueger and Andrews 2011). The methylation status of cytosines in CpG, CHH, and CHG contexts was using Bismark's methylation extractor. The methylation status of both samples was visualized by R using “ggplot2’ package.

### Raw data and code availability

The NGS raw data of total DNA, precipitate, and supernatant portions are available via the National Center for Biotechnology Information (NCBI) with the BioProject number PRJNA1168943. The bisulfite sequencing raw data of supernatant and precipitate portions are available via BioProject number PRJNA1168863 at NCBI. The scripts are available via https://github.com/sarimura639/mitochondrial-DNA-methylation-analysis.

###  

## Supplementary Material

kiaf069_Supplementary_Data

## Data Availability

The data underlying this article are available in National Center for Biotechnology Information (NCBI) at https://www.ncbi.nlm.nih.gov/, and can be accessed with accession number SRR30886772, SRR30886773 for next-generation sequencing (NGS) data of supernatant portion. NGS sequencing data of the precipitate portion can be found under accession numbers SRR30886774 and SRR30886775. NGS sequencing data of total DNA can be found under accession numbers SRR30886776 and SRR30886777. The bisulfite sequencing data of the supernatant and precipitate portion can be found under accession numbers SRR30886319 and SRR30886320, respectively.

## Supplementary data

The following materials are available in the online version of this article.

**Supplementary Figure S1.** The synteny plot between the mitochondrial reference genome and the NUMT sequence, along with the coverage of Illumina short reads from the total, supernatant, and precipitate DNA samples.

**Supplementary Figure S2.** Repetition of the experiment in Fig. 3, SNP calling and allele frequency (AF) calculation of next-generation sequencing (NGS) reads from total, precipitate, and supernatant DNA samples.

**Supplementary Figure S3.** Interpretation of intermediate AF.

## Dive Curated Terms

The following phenotypic, genotypic, and functional terms are of significance to the work described in this paper:

dna Gramene: EO:0007532
dna Araport: EO:0007532
