# Supplementary material for: Mitochondrial DNA and the largest nuclear-mitochondrial DNA in Arabidopsis can be separated by their methylation levels
Source: Plant Physiol. 2025 Feb 20;197(3):kiaf069. doi: 10.1093/plphys/kiaf069 (PMC11879424; doi:10.1093/plphys/kiaf069)
Supplement: kiaf069_Supplementary_Data [file kiaf069_supplementary_data.pdf]

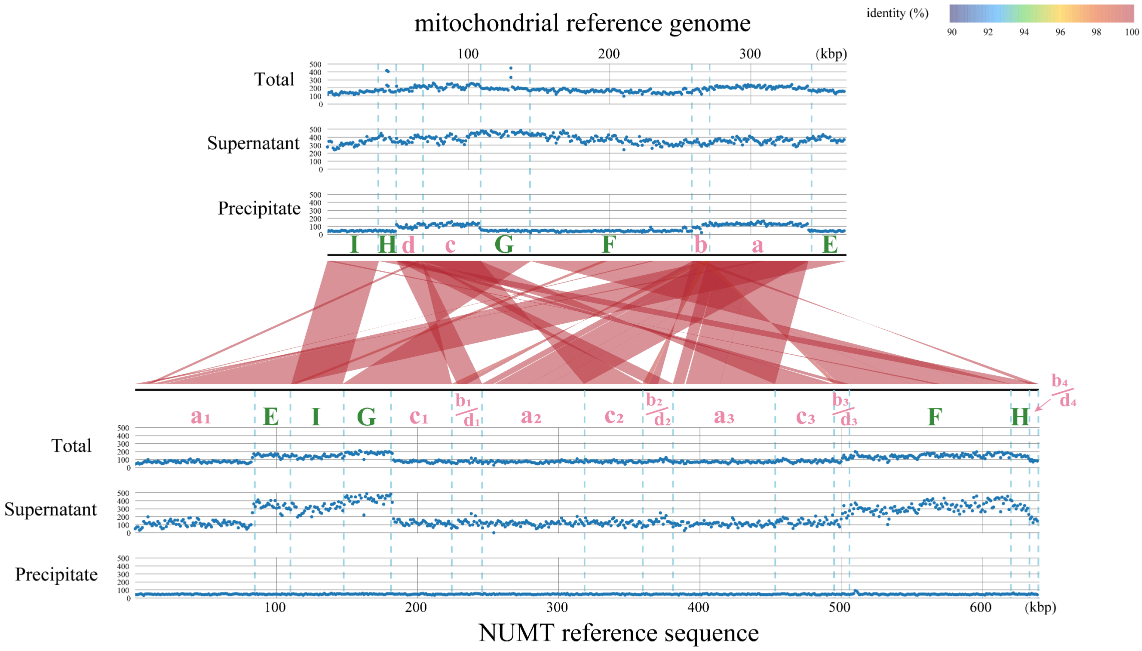

Supplementary Figure S1 The synteny plot between the mitochondrial reference genome and the NUMT sequence, along with the coverage of Illumina short reads from the total, supernatant, and precipitate DNA samples. The upper panel represents the mitochondrial reference genome (BK010421), while the lower panel represents the chromosome 2 NUMT reference sequence (Col-CEN). Green uppercase letters indicate mitochondrial fragments (E~I) that are inserted into a single location on the NUMT (E~I). Pink lowercase letters represent mitochondrial fragments (a ~ d) that are inserted into multiple locations on the NUMT (a_1~a_3, b_1~b_4, c_1~c_3, d_1~d_4). The identity (%) is represented by the colors in the rainbow bar legend, with most lines appearing in red, indicating an overall identity of more than 98%. Reads of total, supernatant and precipitate DNA samples were mapped to mitochondrial reference genome (upper panel) and NUMT reference sequence (lower panel). Total: WGS reads from the total DNA sample. Supernatant: WGS reads from the supernatant sample not captured by the MBD protein-beads. Precipitate: WGS reads from the precipitate sample captured by the MBD protein-beads.

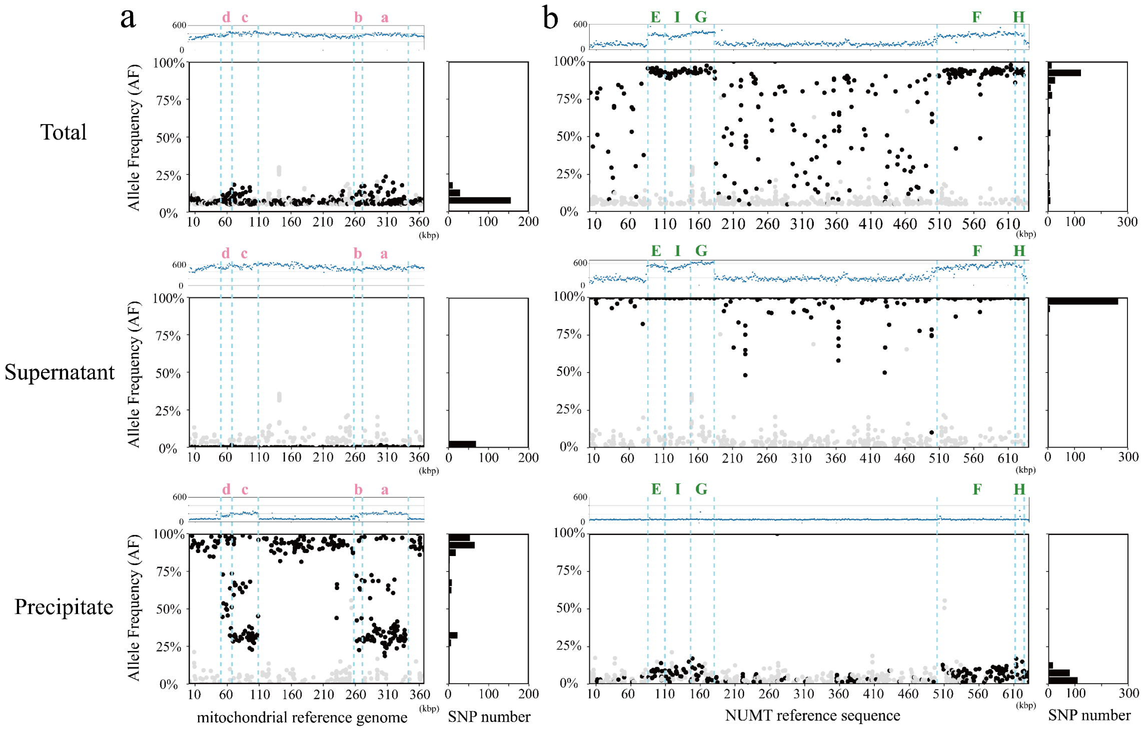

Supplementary Figure S2. Repetition of the experiment in Fig. 3, SNP calling and allele frequency (AF) calculation of NGS reads from total, precipitate and supernatant DNA samples. Reads of total, supernatant and precipitate DNA samples were mapped to **a** mitochondrial reference genome and **b** NUMT reference sequence separately. The black scatter diagrams show the allele positions and frequencies of the SNPs between mitochondrial and NUMT reference sequences, the grey scatters represent sequencing errors and interference reads from chloroplast and NUMT located on chromosomes other than chromosome 2. The blue scatter diagrams at the top of each figure represents the read depth. The total number of SNPs for every 5 % of allele frequency is shown by the bar plots on the right side of each figure. The regions marked by pink lowercase letters correspond to the multi-copy loci in Fig. 2, while the regions marked by green uppercase letters correspond to the single-copy loci in Fig. 2.

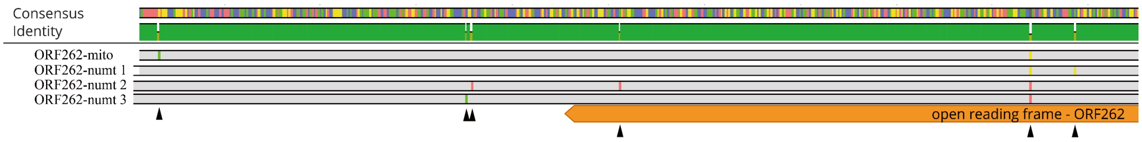

Supplementary Figure S3. Interpretation of intermediate AF. Distribution of SNPs in the mitochondrial (BK010421) and NUMT (Col-CEN) *orf 262* gene regions. Black triangles indicate SNP sites. ORF262-mito: located at positions 269,640–270,159 on the mitochondrial reference genome. ORF262-numt 1: located at positions 11,498–12,017 on the numt reference sequence. ORF262-numt 2: located at positions 245,949–246,468 on the numt reference sequence. ORF262-numt 3: located at positions 381,272–381,791 on the numt reference sequence.
